# Neoadjuvant chemotherapy or upfront surgery in localized pancreatic cancer: a contemporary analysis

**DOI:** 10.1038/s41598-022-17743-6

**Published:** 2022-08-10

**Authors:** Pedro Luiz Serrano Uson Junior, Leonardo Carvalho, Milena Lourenço Coleta Fernandes, Gehan Botrus, Rodrigo de Souza Martins, Elaine Ferreira da Silva, Sarah Silva Mello Batista dos Santos, Leticia Taniwaki, Patrícia Taranto, Ana Carolina Pereira Dutra, João Bosco de Oliveira Filho, Sergio Eduardo Alonso Araujo, Fernando Moura

**Affiliations:** 1 Hospital Israelita Albert Einstein, São Paulo, Brazil; 2 HonorHealth, Phoenix, AZ USA; 3 Center for Personalized Medicine, Hospital Israelita Albert Einstein, São Paulo, Brazil

**Keywords:** Cancer, Biomarkers, Gastroenterology, Medical research, Oncology

## Abstract

Neoadjuvant chemotherapy is considered a new treatment option for potentially resectable pancreatic cancer. However, data are not well established on overall survival and delaying surgery in resectable pancreatic cancer, as well as on those patients that ultimately cannot undergo surgery. We analyzed pancreatic cancer patients treated in a tertiary hospital from January 2016 to December 2020. Patients with resectable stage I and II pancreatic cancer were evaluated regarding surgery, neoadjuvant treatment, and other clinical demographics. The survival function was estimated using the Kaplan–Meier method, and the relationship between the variables of interest and the overall survival (OS) was assessed by adopting the proportional regression Cox models. A total of 216 patients were evaluated. 81 of them with resectable/borderline resectable disease and 135 with unresectable /metastatic disease at diagnosis. Median OS for stage I and II disease were 36 and 28 months, respectively. For resectable pancreatic cancer median OS was 28 months, for borderline resectable pancreatic cancer median OS was 11 months. Median OS for stage III (locally advanced) and stage IV (metastatic) were 10 and 7 months, respectively (p < 0.0001). Median OS of 9 months were obtained for patients with stage I and II that did not undergo surgery compared to 25 months in patients that underwent surgery in any time (p < 0.001). Comparing patients with localized disease, median OS for patients treated with upfront surgery was 28 months, compared to 15 months in patients treated with neoadjuvant approach (p = 0.04). Most patients that did not undergo surgery have decline of performance status or disease progression on neoadjuvant treatment. On multivariable analysis in pancreatic cancer stages I and II, including age, sex, borderline or resectable disease, CA 19–9, positive lymph nodes and neoadjuvant treatment, the surgery was the only factor associated with improved overall survival (p = 0.04). Upfront surgery should still be considered a standard of care approach for resectable pancreatic cancer. Biomarker driven studies and randomized trials with combination therapies are necessary to address neoadjuvant chemotherapy and delaying surgery in purely resectable pancreatic cancer.

## Introduction

Pancreatic cancer incidence is increasing, and it is ranked amongst the five most deadly cancers in the last five years^1,2^. The median overall survival (OS) of localized disease after surgery improved remarkably with the incorporation on modified FOLFIRINOX in the arsenal of adjuvant treatment^3^. Results from the randomized phase III trial comparing modified FOLFIRINOX versus gemcitabine in the adjuvant setting confirmed an impressive median OS of 54.4 months with the combination compared to 35 months in adjuvant gemcitabine group^3^.

New strategies including neoadjuvant chemotherapy have been investigated in the treatment landscape of resectable pancreatic cancer. One of the most awaited randomized trials evaluating this strategy was the PREOPANC trial^4,5^. In this study, a total of 246 patients with localized pancreatic cancer were randomized, 119 were assigned to preoperative chemoradiotherapy, and 127 to immediate surgery^4^. From a total of 133 treated patients with resectable pancreatic cancer, preoperative chemoradiotherapy did not improve median OS (HR 0.79 [95% CI 0.54–1.16]; P = 0.23). Furthermore, median time to distance recurrence, time to local failure, or resectability were not improved for this group of patients with neoadjuvant treatment^4,5^.

A relatively smaller randomized phase II study, SWOG S1505, evaluated neoadjuvant chemotherapy in resectable pancreatic cancer with two contemporary regimens, mFOLFIRINOX (fluoropyrimidine, irinotecan and oxaliplatin) and gemcitabine plus nab-paclitaxel^6^. In that study, the median OS with preoperative mFOLFIRINOX was 23.2 months, and with gemcitabine plus nab-paclitaxel was 23.6 months^6^. Although the study was not powered to compare both regimens, the trial did not demonstrate an improved OS with perioperative chemotherapy compared with historical data from adjuvant trials in resectable pancreatic cancer^3,6^. Neoadjuvant chemotherapy improves OS in anatomically borderline resectable pancreatic cancer^4,5,7^.

Currently, a randomized phase III trial comparing mFOLFIRINOX in the preoperative setting against adjuvant treatment with the same regimen for resectable pancreatic cancer is underway (NCT04340141). One of the biggest issues discussed in neoadjuvant treatment for resectable disease is possible decline of performance status and disease progression. Although some authors advocate that neoadjuvant treatment can select patients better suited for surgery, response to neoadjuvant treatment for selecting biology or surgery could not be entirely accurate^8,9^, considering that some tumors could respond differently to chemotherapy^10,11^. Patients with *BRCA* 1 or 2 mutations have higher responses to platinum-based therapy^10^, and tumors with low *GATA6* expression, basal-like subtype are chemoresistant and they have worse responses to mFOLFIRINOX^11^.

Given the negative results of trials and the lack of biomarker selection in daily practice, we included patients treated in a tertiary hospital with localized resectable pancreatic cancer to evaluate outcomes related to treatment, time of surgery (upfront or after neoadjuvant treatment), and clinical demographics. The aim of this study was evaluating resectable pancreatic cancer patients who ultimately cannot undergo resection.

## Methods

### Patients

Patients with pancreatic ductal adenocarcinoma seen at a tertiary hospital from January 2016 to June 2020 were evaluated. Those individuals with incomplete data for the analysis were excluded from the study. Data were collected on patient’s sex and age, clinical and pathologic stage at diagnosis (8th edition of TNM staging system of pancreatic cancer by AJCC/UICC), neoadjuvant and adjuvant treatment, surgery of the primary tumor, and baseline CA 19-9. For resected pancreatic cancer cases we used the pathological report for TNM staging. For irresectable and metastatic patients the staging was defined by images. Overall survival (OS) was determined by the period between the diagnosis and the date of death or last seen. The definition of resectable disease, borderline disease and locally advanced disease was based on the current National Comprehensive Network Guidelines (NCCN)^12^.

The regimen of choice for neoadjuvant treatment in eligible patients was modified FOLFIRINOX. Dose adjustments for toxic effects were defined by protocol^13^. Patients were restaged with computed tomographic (CT) scans of the chest, abdomen, and pelvis and tumor markers after every 4 cycles of FOLFIRINOX, patients were treated between 8 and 12 cycles before surgery^13^. The research ethics committee of the institution approved the study that followed the existing national standards (CAAE: 81744017.6.0000.0071). Because this was a retrospective study, an exemption of the consent term was requested. All datasets on which the conclusions of the report rely are available upon requesting.

### Statistics

For patients’ baseline characteristics, continuous variables were presented as median (± standard deviation, SD) and categorical variables as percentage. Continuous variables were compared between groups using the F test or Kruskal–Wallis test for parametric and non-parametric values respectively while categorical variables were compared using the chi-square test. Hazard ratios (HRs) and 95% confidence intervals (CIs) were used to describe the association of baseline predictors and overall mortality, after adjustment for baseline differences, using logistic regression analysis with adjustment of baseline differences between resectable versus non-resectable patients. The survival function was estimated using the Kaplan–Meier method, and the relationship between the variables of interest and the survival time was assessed by adopting the proportional chi-square regression Cox models. The results are presented as hazard ratios, 95% confidence interval, and p values. The assumption of proportionality of hazards was assessed by means of hypothesis testing. All analyses were performed using SPSS 11.0 (Chicago, Illinois). Statistical significance was set at a threshold of *P* < 0.05**.**

### Human ethics statement

All experimental protocols were approved by Hospital Israelita Albert Einstein and licensing committee accordingly to national standards (CAAE: 81744017.6.0000.0071). All methods were carried out in accordance with relevant guidelines and regulations. Informed consent is waived due retrospective nature of study and low risk, approved by ethics committee of Hospital Israelita Albert Einstein (CAAE: 81744017.6.0000.0071).

## Results

**Figure 1:**
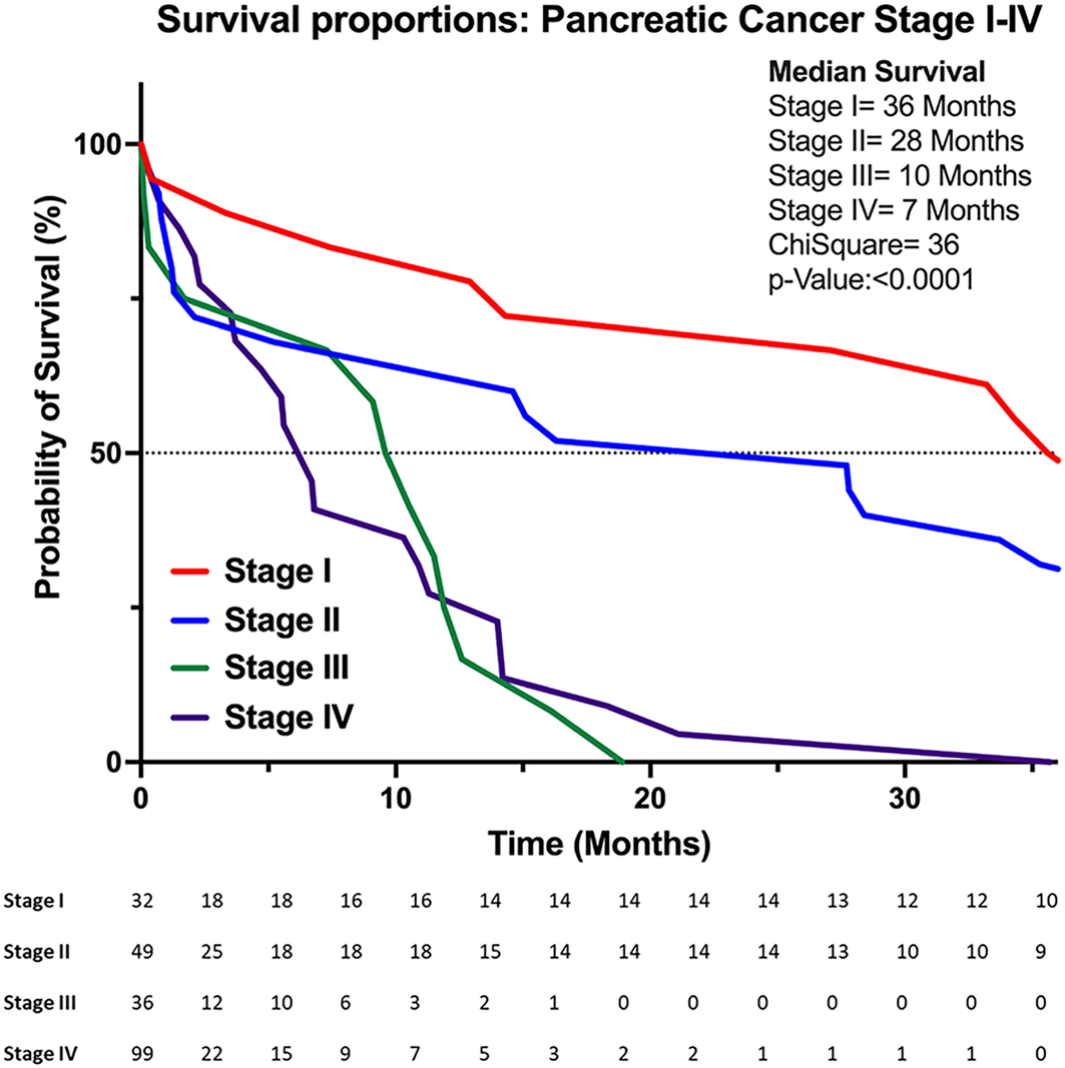
Survival rates by staging.

**Figure 2:**
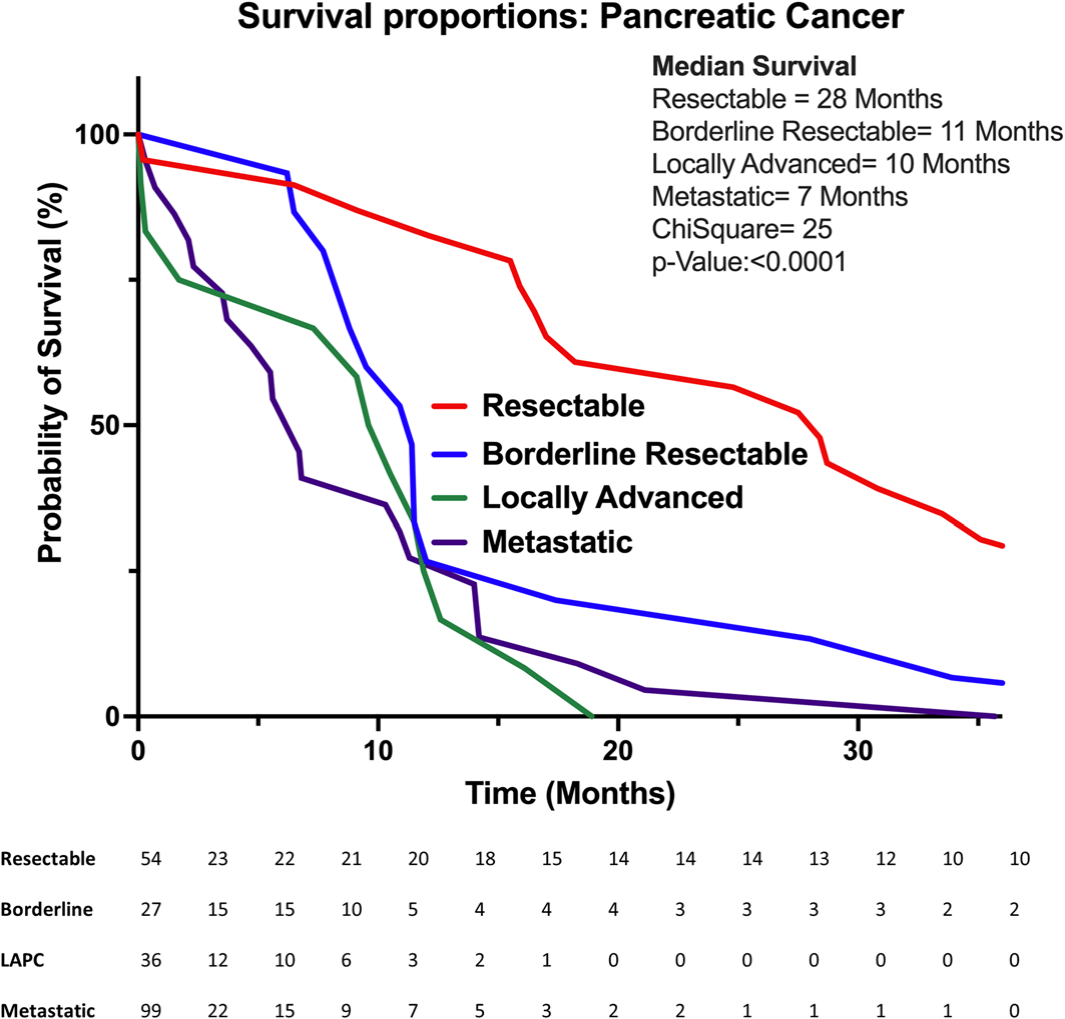
Survival rates by resectability.

The total sample comprised of 216 patients with pancreatic adenocarcinoma, 81 of them belonging to the resectable/borderline resectable group upon diagnosis and 135 to the unresectable /metastatic group at diagnosis. Clinical demographic of all patients is included in Table 1. Overall population median age was 64 years old (32–93). Of patients, half (51%) were men, 37% of them were diagnosed with stage I and II disease. The median overall survival for patients in stage I was 36 months, in stage II was 28 months, in stage III (locally advanced) was 10 months, and in stage IV (metastatic) was 7 months, p < 0.0001 (Fig. 1). For resectable pancreatic cancer median OS was 28 months, for borderline pancreatic cancer median OS was 11 months (Fig. 2). Of 135 patients with advanced pancreatic cancer (stages III and IV) eligible for systemic treatment, 76 (56%) were treated with FOLFIRINOX, 35 (25%) were treated with gemcitabine-based therapies, and one BRCA2 mutated patient received olaparib.

**Table 1:** Clinical demographics of overall population.

Characteristics	Nº (216)
**Age**	
Median	64
Range	32–93
**Sex**	
Male, n(%)	110 (51%)
Female	106 (49%)
**Staging AJCC 8ºedition**	
I	32 (15%)
II	49 (22%)
III	36 (17%)
IV	99 (46%)
**Deceased**	
Yes	138 (64%)
No	78 (36%)
Stages I and II	No. (81)
Resectable pancreatic cancer	54 (67%)
Borderline pancreatic cancer	27 (33%)
**Adjuvant chemotherapy in upfront resected**	No. (41)
mFOLFIRINOX	28 (68%)
Gemcitabine-based	13 (32%)
**Neoadjuvant intent chemotherapy regimens**	No. (34)
mFOLFIRINOX	32 (94%)
Gemcitabine-based	2 (6%)

*AJCC* American Joint Committee on Cancer, *mFOLFIRINOX* fluorouracil, oxaliplatin and irinotecan.

A total of 81 patients were clinically staged as I and II. Demographics characteristics of these patients are shown in Table 2. Median age of patients was 70 years old (40–90), and around half were women, 27 (33%) were defined as borderline resectable by CT scans, 45 (55%) of patients had elevated baseline CA 19–9 (> 37 U/mL), 14 (17%) of patients did not undergo surgery. Thirty-four (42%) patients were submitted to neoadjuvant treatment, in 32 (94%) patients the regimen of choice was modified FOLFIRINOX. Of 67 patients treated with surgery, 41 underwent upfront surgery. Of patients treated with upfront surgery, 28 (68%) were treated with adjuvant modified FOLFIRINOX, other regimens included gemcitabine-based regimens. As suggested, a higher number of patients that did not undergo surgery were borderline resectable cases and had a higher positivity of lymph nodes. Clinical data regarding borderline and resectable patients can be found on supplementary table S1.

**Table 2:** Clinical demographics of localized pancreatic cancer patients.

Characteristics	Surgery: 67 (100%)	No surgery: 14 (100%)
Median age (range)	70 (45–90)	70 (40–90)
Sex male/female	31 (46%)/36 (54%)	7 (50%)/ 7 (50%)
Stage I/II	25 (38%)/42 (62%)	7 (50%)/ 7 (50%)
Borderline resectable (y/n)	17 (25%)/50 (75%)	10 (71%)/4 (29%)
CA19-9 > 37 U/mL (y/n)	36 (54%)/31 (46%)	9 (64%)/5 (36%)
Neoadjuvant treatment intent (y/n)	26 (39%)/41 (61%)	8 (57%)/6 (43%)
Positive lymph nodes (y/n)	33 (49%)/34 (51%)	3 (21%)/11 (79%)

**Figure 3:**
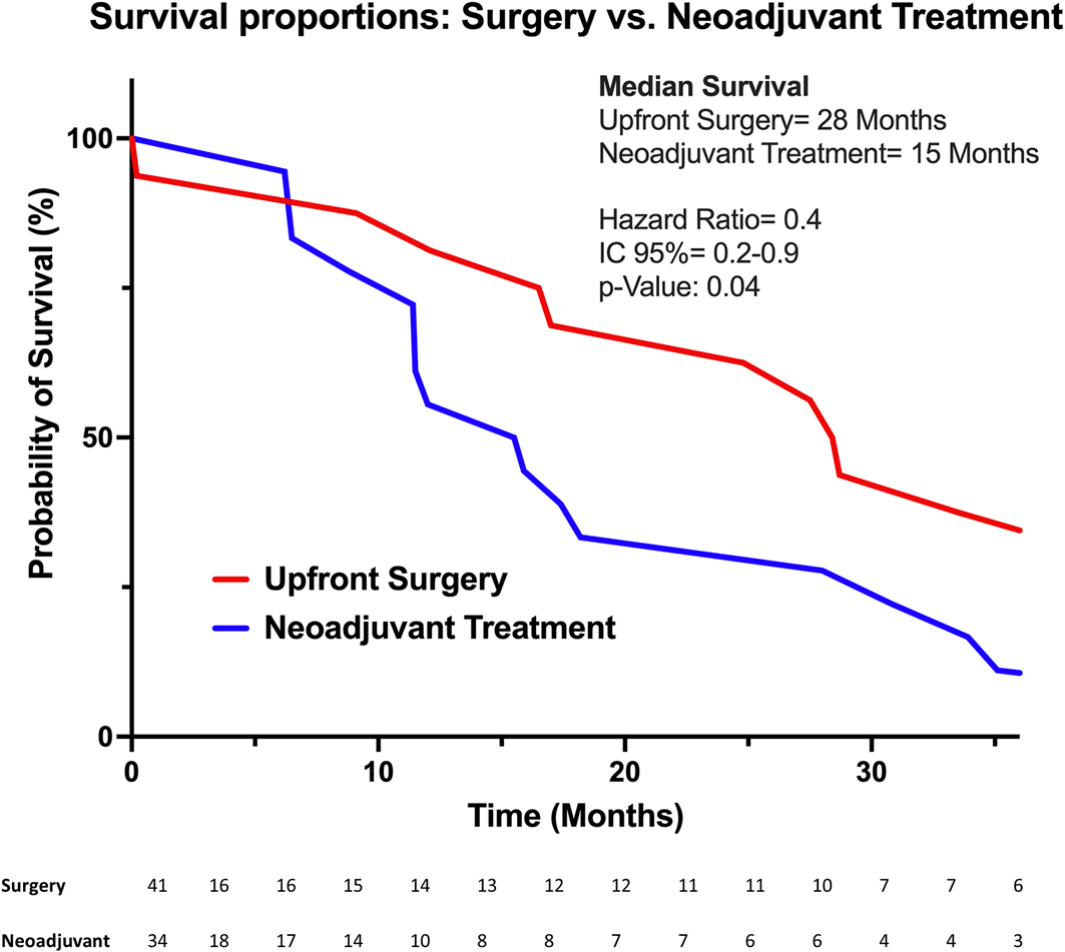
Overall survival in upfront surgery versus neoadjuvant treatment.

Median overall survival for stage I and II patients that underwent surgery was 25 months compared to 9 months in patients that did not undergo surgery, log-rank, (p < 0.001), (Supplementary Fig. S1). From a total of 14 patients that did not undergo surgery, 8 (57%) were not submitted to surgery due to the disease progression or clinical deterioration during neoadjuvant treatment, other causes included baseline poor performance status (5), and patient choice (1). Median OS for patients treated with upfront surgery was 28 months, compared to 15 months in patients treated with neoadjuvant approach (p = 0.04) (Fig. 3). In multivariate analysis, surgery was the only factor that remained statistically significant for overall survival in stage I and II patients (Table 3).

**Table 3:** Multivariable analysis for overall survival in localized pancreatic cancer patients.

Parameter	Hazard ratio	95% CI limits		p value
Sex male vs. female (ref. male)	0.88	0.45	1.72	0.72
Neoadjuvant treatment (y/n) (Ref. yes)	0.77	0.37	1.52	0.47
Stage I vs. II (Ref. Stage I)	1.60	0.59	4.90	0.32
Borderline disease (y/n) (Ref. no)	1.06	0.48	2.32	0.88
Lymph nodes positive (y/n) (Ref. no)	1.26	0.63	2.50	0.50
CA19-9 > 37 U/mL (y/n) (Ref. yes)	0.81	0.41	1.58	0.54
Surgery (y/n) (Ref. no)	0.40	0.17	0.96	0.04

Surgery was the only parameter statistically associated with survival on multivariable analysis. Level of significance p < 0.05.

*Ref* reference.

## Discussion

In this group of patients treated for pancreatic adenocarcinoma in a tertiary center, evaluation of patients with potentially resectable disease showed that surgery remains as one of the most important treatments for localized pancreatic cancer. Median overall survival of patients with stage I and II disease that did not undergo surgery was like patients with locally advanced unresectable disease (9 months versus 10 months). Patients treated with upfront surgery had a median OS of 28 months, patients treated with neoadjuvant intent had median OS of 15 months (p = 0.04). In multivariate analysis surgery remained as the only factor statistically related to improved overall survival (HR 0.4, p = 0.04).

Multiple retrospective studies evaluated neoadjuvant treatment in localized pancreatic cancer, however, most of these studies did not evaluate the patients with localized disease that ultimately were not submitted to surgery. One of the first prospective studies that evaluated surgery in localized disease, and adjuvant gemcitabine, was the CONKO-001 trial^14^. In the study, patients with resectable pancreatic cancer submitted to surgery were randomized to adjuvant chemotherapy with gemcitabine for 6 months or observation. Median overall survival was improved with adjuvant chemotherapy by 10% in 5-years (20.7% versus 10.4%)^14^. In this study, more than 70% of patients were stage T3 and N1 (stage II)^14^. More recently gemcitabine was surpassed by modified FOLFIRINOX, in the randomized phase 3 trial PRODIGE 24^3^. In this study, almost 500 patients were randomized after surgery between the two regimens. Modified FOLFIRINOX was superior against gemcitabine in median disease-free survival (21.6 months versus 12.8 months, p < 0.001), and median overall survival (54.4 months versus 35 months, p = 0.003)^3^. Similar to the CONKO-001, in this study, more than 80% of patients were stage II, with more than 30% of patients submitted to venous resections, including portal vein resections and superior-mesenteric vein resections^3^. The impressive median overall survival obtained with modified FOLFIRINOX determined the new standard of care and the comparator to be achieved in future studies of perioperative chemotherapy in resectable pancreatic cancer.

Growing interest in neoadjuvant chemotherapy in both resectable pancreatic cancers and locally advanced disease combined with retrospective observations led to the development of randomized trials evaluating the strategy. As discussed before, OS results presented from the PREOPANC trial in the subgroup of resectable patients were not superior with neoadjuvant treatment with gemcitabine plus radiotherapy, compared to adjuvant treatment, HR for resectable disease 0.79 (95% CI, 0.54 to 1.16); P = 0.23)^4,5^. In the group of anatomically borderline resectable pancreatic cancer, neoadjuvant treatment improved overall survival by 4 months (17.6 months versus 13.2 months), improved local failure rates and R0 rate (79% versus 13%)^4^. It is important to mention a limitation of the study, such as modified FOLFIRINOX that was not standard chemotherapy regimen when it started^4^. However, in the four-arm prospective ESPAC-5F trial, modified FOLFIRINOX had the best survival at one year as neoadjuvant treatment for borderline resectable pancreatic cancer against upfront surgery, capecitabine plus radiotherapy or gemcitabine and capecitabine, but no difference in resection rate was observed^7^.

In our data, more than 90% of patients treated with neoadjuvant chemotherapy were treated with modified FOLFIRINOX as the perioperative chemotherapy regimen of choice. Surgery more than doubled the median overall survival of the eligible patients. Furthermore, in localized disease, patients treated with upfront surgery had higher OS than patients treated with neoadjuvant intent. Differently for selecting the best perioperative regimen for localized pancreatic cancer, our objective with this analysis was also to identify motivations for delaying surgery. Three quarters of patients not submitted to surgery were due disease progression or clinical deterioration during neoadjuvant treatment. Interestingly, in the SWOG S1505 trial, patients with resectable pancreatic cancer were treated with two combinations of neoadjuvant chemotherapy, modified FOLFIRINOX and gemcitabine plus nab-paclitaxel^5^. In the study, around 70% of the patients underwent resection after neoadjuvant treatment, and less than half completed the treatment with postoperative chemotherapy^5^. The median overall survival of both cohorts were around 23 months, not superior to adjuvant modified FOLFIRINOX in historical data^3^. Furthermore, both arms did not reach pre specified efficacy measurements^5^. However, patients included in the PRODIGE 24 trial are highly selected patients considering that they were selected for surgery and adjuvant modified FOLFIRINOX, so the historical comparison raised in this discussion needs to be taken with caution. After all these results neoadjuvant treatment in resectable pancreatic cancer should be further evaluated in larger randomized trials before to be considered a universal approach to all patients.

Multiple strategies are being developed to improve outcomes in resectable pancreatic cancer. Selecting patients based on germline testing could improve responses to neoadjuvant treatment. Patients with germline *BRCA1/2* and *PALB2* mutations have higher responses to platinum-based chemotherapy regimens including oxaliplatin and cisplatin^7,15^. Furthermore, these patients have benefit with the addition of poly adenosine diphosphate-ribose polymerase inhibitors (PARPi)^16^. Olaparib, a PARPi, is being evaluated in adjuvant setting in a randomized phase II trial for resectable pancreatic cancer with germline *BRCA1/2* or *PALB2* mutation after perioperative chemotherapy (NCT04858334). Another way to select patients that most benefit for neoadjuvant chemotherapy would be incorporating biomarkers selection, one example is the evaluation of *GATA6* expression^8,17^. Tumors that express *GATA6*, either detected by RNA sequencing or immunohistochemistry, are defined as classical subtype, and tend to have higher responses to FOLFIRINOX, in opposite of the basal-like subtype, which have low expression or do not express *GATA6*, and have worse outcomes^8,17,18^. Finally, circulating cell-free tumor DNA could be a factor to stratify patients with better outcomes in future prospective trials, and possibly select patients that have benefit to neoadjuvant approaches^19–22^. Although biomarker selection could improve the outcomes of neoadjuvant treatment, a randomized phase III trial with no biomarker selection is ongoing, with preoperative versus adjuvant modified FOLFIRINOX in resectable pancreatic cancer (NCT04340141). Smaller nonrandomized studies evaluating new strategies including neoadjuvant treatment based on the molecular subtype (classical vs. basal-like) (NCT04683315) and adaptive modification of neoadjuvant treatment based on response are underway (NCT03322995).

Based on all the trials with modern chemotherapy regimens, so far, there is no clear benefit of neoadjuvant treatment in purely resectable pancreatic cancer. This contemporary analysis has some limitations, including small sample size and retrospective nature of data collection. This analysis should be better evaluated in a prospectively manner. Considering that all patients treated in the period were included for analysis, a propensity score matched analysis would not be feasible as well. The results of this study should be evaluated as a hypothesis generation. Outside clinical trials the choice of neoadjuvant treatment should include germline testing and possible biomarker tumor testing for better selection of patients that ultimately would lead to higher responses. Future strategies for better selection of patients who should undergo neoadjuvant treatment would include biomarker selection and/or stratification with ctDNA.

## Supplementary Information

Supplementary Information 1.

## Data Availability

The datasets generated and/or analyzed during the current study are not publicly available due National General Data Law Protection (LGPD) but are available from the corresponding author on reasonable request.
