# Supplementary material for: Neoadjuvant chemotherapy or upfront surgery in localized pancreatic cancer: a contemporary analysis
Source: Sci Rep. 2022 Aug 10;12:13592. doi: 10.1038/s41598-022-17743-6 (PMC9365816; doi:10.1038/s41598-022-17743-6)
Supplement: Supplementary file 1 — Supplementary Information 1. [file 41598_2022_17743_MOESM1_ESM.docx]

| Localized disease (N=81) | Resectable disease (N=54) | Borderline disease (N=27) |
| --- | --- | --- |
| Age |  |  |
| -Median | 70 | 65 |
| -Range | 45-90 | 40-90 |
| Sex (male/female) | 30/24 | 16/11 |
| Ca 19-9 (>37 U/ml) (yes/no) | 28/26 | 17/10 |
| Neoadjuvant treatment (yes/no) | 15/39 | 19/8 |
| Type of neoadjuvant treatment |  |  |
| - Neo. Chemotherapy alone | 10 | 15 |
| - Chemoradiotherapy | 5 | 4 |
| Adjuvant treatment | 33 | 17 |
| Duration of perioperative systemic treatment |  |  |
| - Median (months) | 4 | 4 |
| - Range | 2-6 | 2-6 |
| Surgery (yes/no) | 50/4 | 17/10 |
| Deceased (yes/no) | 23/31 | 15/12 |

**Supplementary table 1: Clinical demographics of resectable and borderline resectable patients.**

**Supplementary figure 1: OS in patients submitted to surgery versus non-surgery**

**
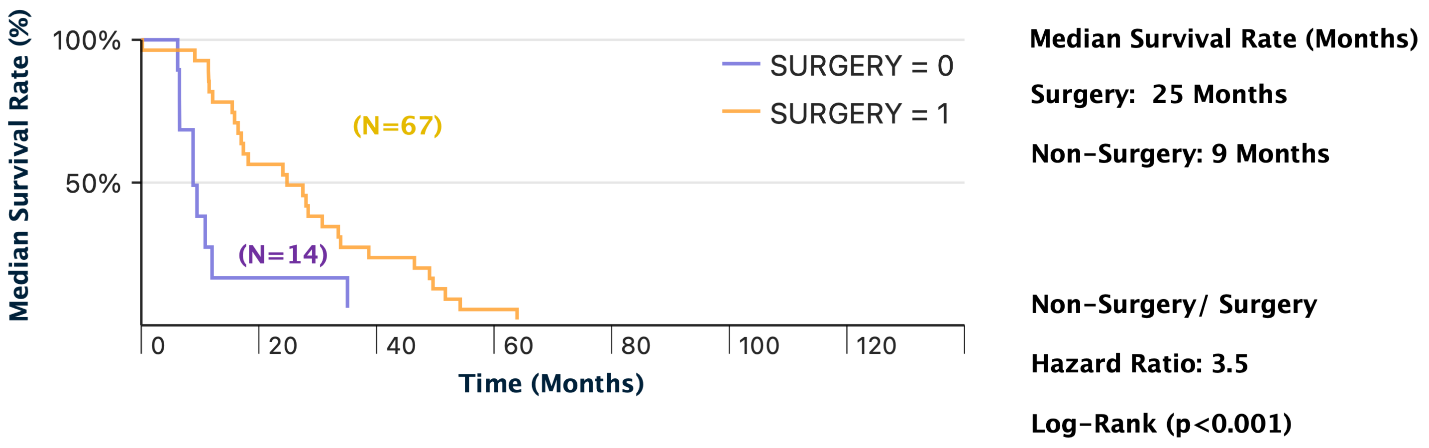
**
